# IV t-PA Influences Infarct Volume in Minor Stroke: A Pilot Study

**DOI:** 10.1371/journal.pone.0110477

**Published:** 2014-10-28

**Authors:** Kersten Villringer, Ulrike Grittner, Lars-Arne Schaafs, Christian H. Nolte, Heinrich Audebert, Jochen B. Fiebach

**Affiliations:** 1 Academic Neuroradiology, Department of Neurology and Center for Stroke Research, Charité, Berlin, Germany; 2 Department for Biostatistics and Clinical Epidemiology and Center for Stroke Research, Charité, Berlin, Germany; 3 Department of Neurology and Center for Stroke Research, Charité, Berlin, Germany; Weill Cornell Medical College, United States of America

## Abstract

**Background:**

There is an ongoing debate whether stroke patients presenting with minor or moderate symptoms benefit from thrombolysis. Up until now, stroke severity on admission is typically measured with the NIHSS, and subsequently used for treatment decision.

**Hypothesis:**

Acute MRI lesion volume assessment can aid in therapy decision for iv-tPA in minor stroke.

**Methods:**

We analysed 164 patients with NIHSS 0–7 from a prospective stroke MRI registry, the 1000+ study (clinicaltrials.org NCT00715533). Patients were examined in a 3 T MRI scanner and either received (n = 62) or did not receive thrombolysis (n = 102). DWI (diffusion weighted imaging) and PI (perfusion imaging) at admission were evaluated for diffusion - perfusion mismatch. Our primary outcome parameter was final lesion volume, defined by lesion volume on day 6 FLAIR images.

**Results:**

The association between t-PA and FLAIR lesion volume on day 6 was significantly different for patients with smaller DWI volume compared to patients with larger DWI volume (interaction between DWI and t-PA: p = 0.021). Baseline DWI lesion volume was dichotomized at the median (0.7 ml): final lesion volume at day 6 was larger in patients with large baseline DWI volumes without t-PA treatment (median difference 3, IQR −0.4–9.3 ml). Conversely, in patients with larger baseline DWI volumes final lesion volumes were smaller after t-PA treatment (median difference 0, IQR −4.1–5 ml). However, this did not translate into a significant difference in the mRS at day 90 (p = 0.577).

**Conclusion:**

Though this study is only hypothesis generating considering the number of cases, we believe that the size of DWI lesion volume may support therapy decision in patients with minor stroke.

**Trial Registration:**

Clinicaltrials.org NCT00715533

## Introduction

Thrombolysis has become standard therapy in the treatment of acute ischemic stroke and is used within a time frame of ≤4.5 hours in Europe since ECASS 3 [1]. However, uncertainty remains when it comes to treating patients with minor stroke or rapidly improving symptoms, especially considering the side effects of t-PA with an increased risk of intracerebral or extracranial haemorrhages [2].

Moreover, there is disagreement regarding the outcome in minor stroke patients with reports of excellent outcome at 3 months follow-up without any intervention [3] but also poor outcome reported in nearly one third when withholding thrombolysis [4], [5].

MRI has been suggested to identify patients with minor stroke as well as point out those who might suffer a recurrent stroke and might therefore benefit from thrombolysis. Rajajee et al. [6] and others [7] found vessel pathology to be predictive for recurrent stroke in transient ischemic attacks (TIA) and minor stroke patients as well as for outcome. Vessel pathology was also associated with clinically beneficial treatment effects. However, the included patients showed signs of moderate to severe stroke in an extended time window of <9 hours from symptom onset to treatment [8].

This leaves minor stroke patients who show no vessel pathology but are considered as possibly benefitting from thrombolysis.

It also has been shown that a mismatch volume of more than 10 ml is predictive of lesion progression and early neurological deterioration [9], though, perfusion imaging cannot always be obtained for instance due to renal failure.

Furthermore, to save time, especially at the end of the mandated time window it might be necessary to shorten the MRI imaging protocol to haemorrhage sensitive sequences (T2*) and diffusion weighted imaging (DWI).

Therefore, we aimed to evaluate the potential of DWI at admission in patients with low/moderate NIHSS in facilitating therapy decision towards thrombolysis by comparing minor stroke patients who did and did not receive t-PA.

## Patients and Methods

**Figure 1:**
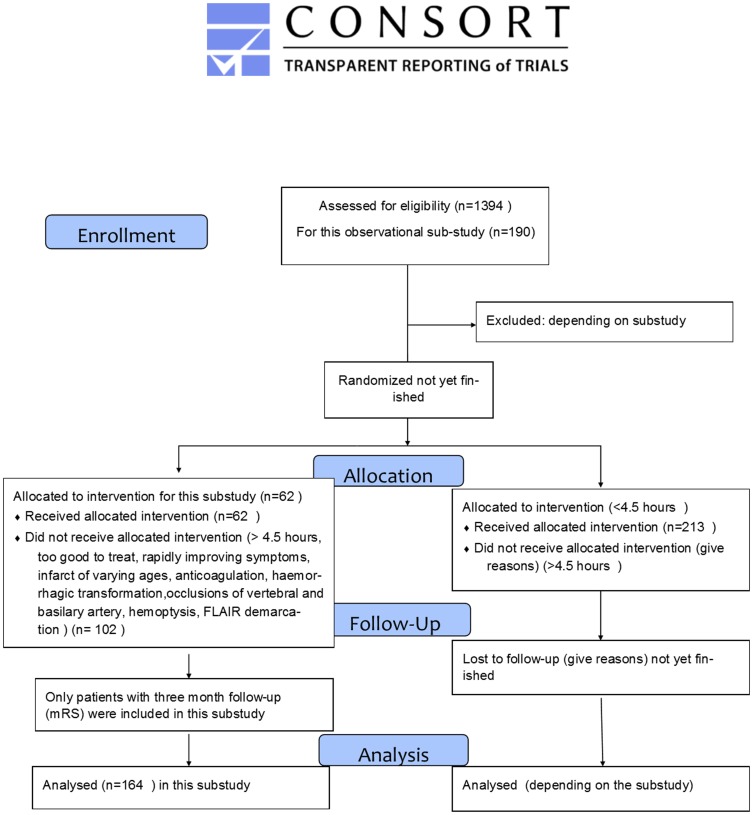
CONSORT 2010 Flow Diagram.

The protocol for this trial and supporting CONSORT checklist are available as supporting information; see Checklist S1, Protocol S1 and Figure 1.

From 11/2008 until 6/2012 we included 190 patients with ischemic stroke from the observational 1000+ study, registered at clinicaltrials.org NCT00715533.

### Ethics statement

Written informed consent was obtained from all patients. The study design (1000+ study) was approved by the ethics committee of the Charité - Universitätsmedizin, Berlin (EA4/026/08).

### MRI Examination

Data acquisition was performed on a 3 Tesla MRI scanner (Tim Trio, Siemens AG, Erlangen, Germany). The imaging protocol included a diffusion weighted imaging (DWI) (TR/TE/FOV/matrix: 7600 ms/93 ms/230 mm/192×192, slice thickness 2.5 mm, b = 1000 s/mm^2^, 6 different directions), bolus – track perfusion imaging (PI) (TR/TE/flip angle/FOV/matrix: 1390 ms/29 ms/60°/230 mm/128×128, slice thickness 5 mm), a fluid-attenuated inversion recovery sequence (FLAIR) (TR/TE/TI/flip angle/FOV/matrix: 8000 ms/100 ms/2370.5 ms/130°/220 mm/256×256, slice thickness 5 mm) and a 3 dimensional time-of-flight magnetic resonance angiography (MRA) (TR/TE/flip angle/field of view/slice thickness: 22 ms/3.9 ms/20°/162*200/0.7 mm). For the perfusion measurements a bolus of 5 ml Gadovist was administered, followed by a saline flush both at a flow rate of 5 ml/s.

Postprocessing of perfusion images was performed with the Siemens standard software package resulting in regional cerebral blood flow (rCBF), regional cerebral blood volume (rCBV) and mean time-to-peak (MTT) maps with the latter used for further analysis. Regions of interests (ROI) were manually drawn on DWI, MTT – maps and FLAIR day 6 images. Lesion volumes were calculated from ROIs, acute absolute mismatch volume (MTT volume – DWI volume) and mismatch ratio (MTT volume/DWI volume) were assessed.

### Statistical analysis

All continuous variables were checked for normal distribution (histogram check, absolute skewness-value <1) before using parametric tests. Otherwise non-parametric statistical tests were applied or the variables were transformed before analysis to reduce skewness in distribution. The Mann-Whitney test was employed to compare differences between groups for quantitative non-normally distributed outcomes. To assess differential treatment ((iv) t-PA) effects for patients with different DWI volume on admission with regard to FLAIR volume on day 6, a regression analysis was performed using log-transformed values for lesion volumes with log-transformed FLAIR volume as outcome. Log-transformation was performed to overcome the skewed distributions. A regression analysis was also used to evaluate the association between vessel status and FLAIR volumes on day 6. A level of α = 0.05 was considered significant. No adjustment for multiple testing was applied.

For exploratory purposes we used a median split of 0.7 ml to assess the effect of thrombolysis on DWI and FLAIR volumes. All statistical analyses were performed using SPSS version 20.

## Results

The inclusion criteria for this study were as follows:

Visible lesions on DWI at admission, since it could be demonstrated that only 4 out of 299 patients with negative DWI images had perfusion deficits, indicating ischemia [10]. Further criteria were time interval between symptom onset and MRI examination ≤9 hours in view of interventional studies like AXIS or DIAS III/IV, as well as mild to moderate stroke with NIHSS up to 7 [11], [12] with or without t-PA therapy.

One-hundred-and-sixty-four out of 190 patients with minor/moderate stroke were considered for further analysis.

The reasons for exclusion were incomplete examinations (n = 7), faulty entry to database (n = 5), unknown symptom onset (n = 5), onset>9 hours (n = 4), mechanical thrombus extraction (n = 2), no visible DWI lesion (n = 1), one haemorrhage and one patient with postictal edema.

The reasons for patients within the time window of less than 4.5 hours (n = 50) for not receiving t-PA were as follows:

Rapidly improving symptoms (n = 24), patients considered “too good” to treat (n = 6), infarcts of varying ages (n = 4), oral anticoagulation (n = 3), haemorrhagic transformation (n = 1), one patient had occlusions of both vertebral arteries and the basilary artery, one had a clear demarcation on FLAIR and the last one had haemoptysis. In 9 patients no further information on the reasons for exclusion from t-PA therapy could be obtained.

Detailed patient characteristics are given in table 1.

**Table 1:** Patients characteristics.

n = 164	NIHSS 0–7 iv t-PA	NIHSS 0–7 non iv t-PA	p^a^
n	62	102	
**gender** (female) n (%)	26 (41.9%)	38 (37.3%)	0.621
**age** (years)	71 (63–77)	70 (62–78)	0.888
**onset – MRI** (minutes)	105 (79–147)	233 (122–382)	<0.001
**DWI** (ml)	0.8 (0.3–3.8)	0.7 (0.2–2.3)	0.279
**relMTT** (ml)	12.0 (0.3–60.2)	6.4 (0.0–35.9)	0.220
**MM volume** (ml)	7.1 (−0.1–49.8)	3.5 (−0.1–34.2)	0.545
**MM ratio**	3.1 (0.0–24.6)	3.7 (0.0–16.7)	0.939
**FLAIR day6** (ml)	3.2 (0.7–7.0) (n = 46)	2.3 (0.6–8.9) (n = 72)	0.623
**mRS day 90**	1 (0–2) (n = 62)	1 (0–2) (n = 102)	0.577
**NIHSS day1**	4 (2–5) (n = 62)	2 (1–4) (n = 102)	0.002
**NIHSS day6**	1 (0–2) (n = 51)	1 (0–2) (n = 88)	0.57

a Mann - Whitney for differences in thrombolysis versus non intravenous (iv) t-PA

Age, onset, volumes, NIHSS, mRS are given as median, interquartile range (IQR)

iv t-PA  =  intravenous tissue plasminogen activator, ml  =  millilitre, MM  =  mismatch

There were no significant differences in baseline variables between patients treated with iv t-PA and those not treated, with the exception of time between onset and MRI examination with shorter delays in treated patients, median:105 minutes (IQR 79–145 minutes) compared to 233 minutes (IQR 122–380 minutes) in untreated patients (p<0.001). There was also a difference in NIHSS values at admission: median NIHSS 4 (IQR 2–5) in iv t-PA treated patients compared to 2 (IQR 1–4) in the non iv t-PA group (p = 0.002). No significant difference for mRS at 3 months follow-up was found between groups (median 1 (IQR 0–2)) (p = 0.577).

**Figure 2:**
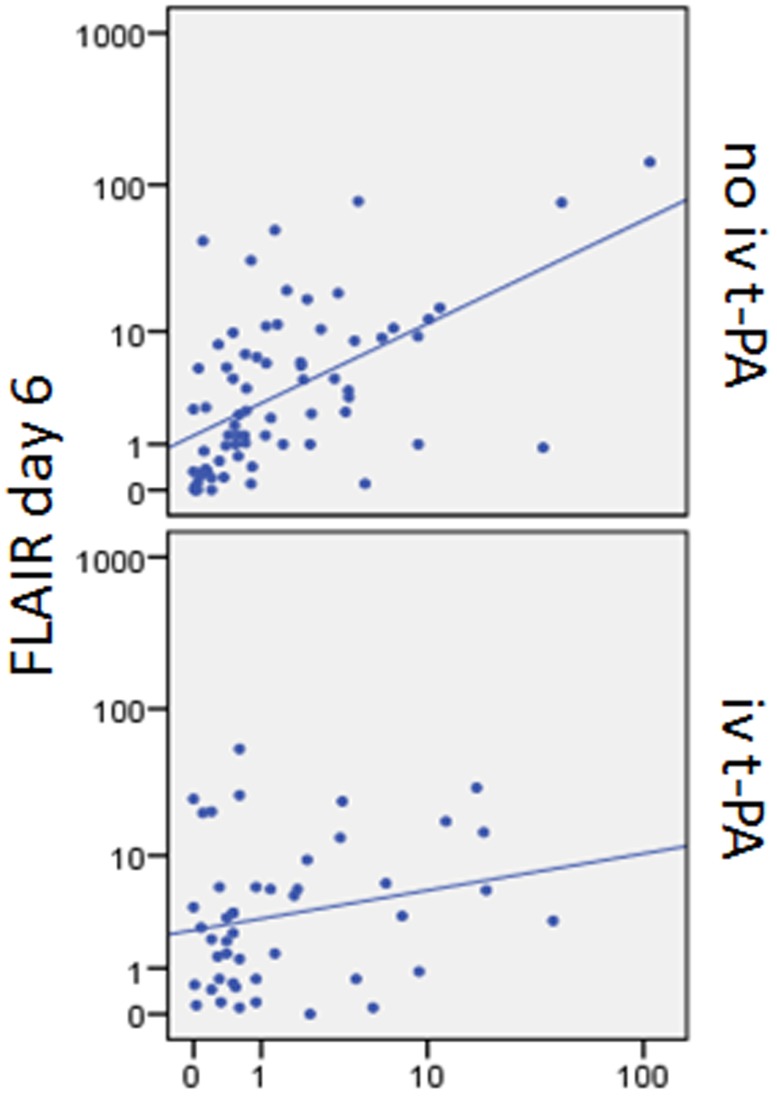
Association between DWI volume day 1 and FLAIR volume day 6.

**Figure 3:**
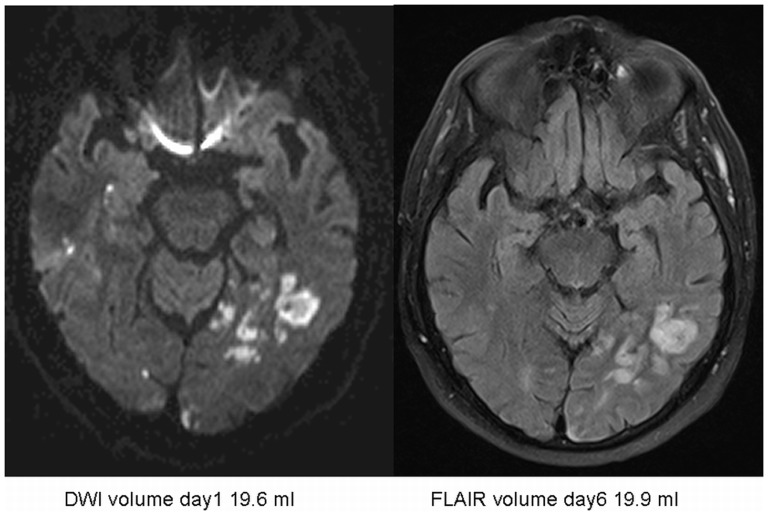
Lesion volume development in minor/moderate stroke after iv t-PA.

**Figure 4:**
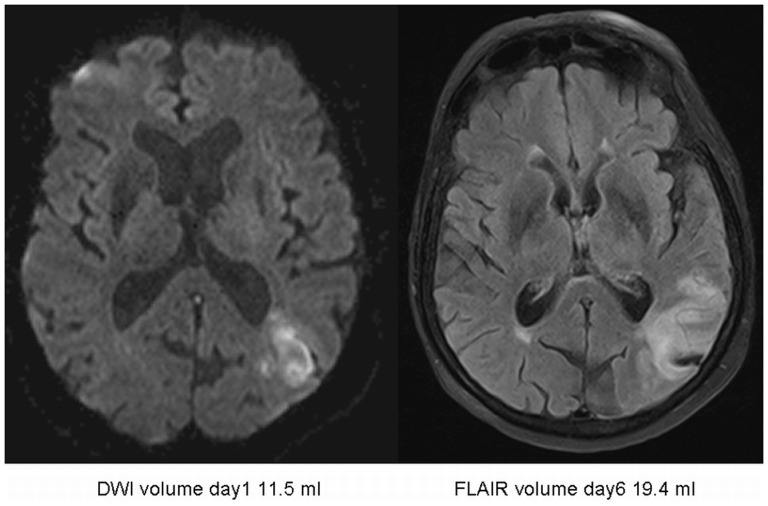
Lesion volume development in minor/moderate stroke without therapy.

Though no significant differences between the median DWI volume at admission and FLAIR volume on day 6 could be demonstrated, the regression analysis showed a significant interaction of DWI volume and thrombolysis on FLAIR day 6 volumes as outcome parameter (p = 0.021) (figure 2, table 2) indicating differential treatment effects for those with smaller DWI volumes compared to those with larger DWI volumes. To further explore this result we performed a median split [13] at 0.7 ml baseline DWI volume and compared infarct growth in patients who received t-PA and in those who did not (table 3). The largest increase in lesion volume was found in those patients with large admission DWI volumes who did not receive t-PA (median difference: 3.01 ml, IQR: −0.35–9.28 ml). The smallest lesion growth could be detected in patients with large DWI volume who received t-PA (median difference: 0 ml, IQR −4.10–5.00 ml) (figure 3, 4). No significant interaction between vessel status at baseline and outcome could be demonstrated for either groups (iv t-PA treated and untreated) (p = 0.928). Analysing both groups separately those patients with occlusions, not receiving iv t-PA showed a tendency towards larger FLAIR day 6 volumes compared to patients with no vessel pathology. Contrary, in patients with vessel occlusion treated with iv t-PA FLAIR volumes on day 6 tended to be smaller compared to those without vessel pathology.

**Table 2:** Linear regression analysis for FLAIR day 6 (log-tranformed) (n = 118, adjusted r^2^ = 0.23).

	β (se)	p
constant	0.35 (0.10)	0.001
NIHSS day 1 (log-transformed)	0.20 (0.13)	0.113
DWI volume (log-transformed)	0.64 (0.13)	<0.001
iv t-PA (versus non iv t-PA)	0.001 (0.13)	0.997
Interaction t-PA*DWI volume	−0.44 (0.19)	0.021
Vessel status (reference: no pathology)		
occlusion	0.01 (0.14)	0.928
stenosis	−0.13 (0.16)	0.416

**Table 3:** Lesion volume changes of DWI volume and FLAIR day 6 by DWI volume categories and t-PA therapy.

	low DWI volume (<0.7 ml)		high DWI volume (≥0.7 ml)	
	iv t-PA (n = 25)	non iv t-PA (n = 32)	iv t-PA (n = 21)	non iv t-PA (n = 40)
**DWI volume (ml)** median, interquartile range	0.32 (0.15–0.50)	0.18 (0.04–0.48)	3.60 (1.55–10.70)	2.25 (1.10–4.70)
**FLAIR volume day 6 (ml)** median, interquartile range	2.1 (0.58–4.92)	0.74 (0.20–2.33)	5.50 (0.80–11.35)	5.85 (2.02–12.03)
**Difference FLAIR volume day 6/DWI volume (ml)** median, interquartile range	1.90 (0.33–4.77)	0.39 (0.04–2.15)	0.00 (−4.10–5.00)	3.01 (−0.35–9.28)

iv  =  intravenous, n  =  number, ml  =  millilitre.

## Discussion

In this pilot study we found a significant association between infarct volume changes and thrombolytic treatment. Treatment effect in terms of smaller infarct growth was stronger in patients with larger DWI volumes compared to those with smaller DWI volumes. This argues for baseline DWI volume as useful parameter in assisting therapy decision towards or against thrombolysis in minor stroke patients.

There is still considerable uncertainty regarding therapy decision resting upon clinical scales like the NIHSS alone. Many attempts have been undertaken to assess non-isolated symptoms as defining variables in minor stroke (NIHSS ≤6) [14], different combinations of NIHSS items and cut-offs [15] or defining key symptoms like language impairment and distal paresis, which may justify the use of thrombolysis [16].

There have also been many efforts to establish imaging variables capable of predicting deficit and severity of disability due to stroke, therefore aiding therapy decisions. Among those the most common imaging parameter is the perfusion – diffusion mismatch to detect potentially salvageable tissue. Recently, for minor stroke, a volume of mismatch above 10 ml was found to be associated with infarct growth and neurological deterioration [9]. However, there are often situations which impede or even prevent the application of contrast media as well as technical problems like motion artefacts complicating the analysis of perfusion maps. In more severe stroke, there is evidence, that mismatch severity may be relevant for selecting patients suitable for thrombolytic treatment [17] with a mismatch ratio of 2.6 proving the highest sensitivity (90%) and specificity (83%) [18]. In minor stroke this observation was not yet established, but may also apply.

Other studies describe correlations between acute DWI and PI lesion volumes and acute and chronic stroke scales [19]–[21] with the most powerful predictive parameter for recanalisation being baseline NIHSS score <15 and lower absolute TPP. Furthermore baseline DWI lesion volume was described to be highly correlated with final infarct size (p<0.0001) on day 60 [22].

Another strong relationship was reported between infarct extent and location providing a good estimate of neurological deficit within 3 months of stroke onset [23]. However, for the acute/subacute phase further evidence is required.

The association between the extent of infarct growth and outcome in patients with moderate/severe stroke symptoms has already been proven [24], as well as the contribution of vessel abnormalities [7]. Though, no attempts have been made to assess the prediction of DWI baseline infarct volume on outcome in t-PA treated and untreated minor stroke patients.

As a novel finding in our study, infarct growth was attenuated by thrombolysis in patients with larger DWI infarcts. However, within our small cohort the median mRS at day 90 did not differ significantly in treated and untreated patients, hence, no clear prediction regarding long term outcome can be drawn. Likewise, Urra et al., in a study cohort of 203 patients did not find significant differences in mRS on day 90, but did find a decrease in the mRS in the ordinal regression analysis [25] indicating an improved long term outcome too.

The lack of a significant difference in long term outcome may also be explained by the limited capacity of the mRS in depicting post stroke disorders like fatigue, depression [26] or cognitive problems. These symptoms may be just as likely in low NIHSS stroke patients and can considerably influence quality of life. Hence, other instruments assessing quality of life are required in minor stroke patients [27].

We found no association between vessel status and outcome, but untreated minor stroke patients with vessel occlusions showed, at least for the short term, an increase in infarct volume compared to iv t-PA treated patients, therefore pointing to the effect of thrombolysis. This observation is shared by others [22], [28], however, in cohorts of more severe stroke patients compared to our study cohort.

The limitation of our study is clearly the small number of patients, indicating that this study is underpowered to sufficiently predict long term outcome and thus may be considered as only hypothesis generating. Using a median split of baseline DWI volume at 0.7 ml seems to be arbitrary at the moment, however, in dichotomizing the data the influential effect of t-PA treatment could be illustrated. Though, using this method a loss or overestimation of effect size and power in case of bivariate relationships or analysis of independent variables must be kept in mind [13]. Moreover, the extended time window of up to 9 hours after symptom onset may negatively affect lesion volume evolution. This may account for the large increase in large DWI lesion volumes in untreated patients. Another source of bias represents the decision for t-PA treatment based upon clinical reasons alone. Thus, further prospective studies to support or reject our results are required.

## Conclusions

The size of baseline DWI infarct volumes can support therapy decision for iv t-PA in minor stroke, in addition to findings of perfusion imaging and NIHSS score. Though, regarding the number of cases, this study must be considered as only hypothesis generating.

## Supporting Information

Checklist S1 **CONSORT checklist.**
(DOC)

Protocol S1 **Trial protocol.**
(DOCX)
